# Impact of Early Surgical Intervention of Plastic Surgeons on the Prognosis of Necrotizing Soft Tissue Infection

**DOI:** 10.7759/cureus.19382

**Published:** 2021-11-08

**Authors:** Ai Yokoyama, Chikara Takase

**Affiliations:** 1 Department of Plastic and Reconstructive Surgery, Yokosuka General Hospital Uwamachi, Yokosuka, JPN

**Keywords:** early debridement, outcome analysis, plastic and reconstructive surgery, plastic surgery, infection, fournier’s gangrene, necrotizing fasciitis, necrotizing soft tissue infection

## Abstract

Background

Necrotizing soft tissue infection (NSTI) is a rare, severe bacterial infection that causes rapidly progressive soft tissue necrosis from the skin to the muscle. The gold standard for treating NSTI is a prompt diagnosis, early surgical debridement of necrotic tissue, and antimicrobial therapy. This study investigated the relationship between the involvement of plastic surgeons and the clinical course of NSTI cases treated at Yokosuka General Hospital Uwamachi.

Methodology

This study involved 28 patients with NSTI who were treated at Yokosuka General Hospital Uwamachi. Patient background, outcomes (mortality and amputation), and days to the first surgery were compared in the early and nonearly plastic surgery intervention groups. Moreover, the duration of treatment was also compared in surviving patients. Differences between the two groups were analyzed using Fisher’s direct probability test, Mann-Whitney U test was used for comparison of continuous variables between the two groups, and Spearman’s rank correlation analysis was used for the bivariate correlation coefficient. The significance level was set at <5%.

Results

There were eight and 20 patients in the early and nonearly plastic surgery intervention groups (14 in later intervention and six in nonintervention), respectively. A difference in the median number of days to the first surgery between the early (zero days) and the nonearly (two days) intervention groups was significant (p = 0.002). In the survival groups, the median treatment duration in the early (n = 8) and nonearly (n = 13) intervention groups was 44 and 82 days, respectively, which was significantly shorter in the early intervention group (p = 0.003).

Conclusions

The number of days until the first surgery and the length of the treatment period were significantly shorter in the early plastic surgery intervention group than in the nonearly intervention group.

## Introduction

Necrotizing soft tissue infection (NSTI) is a rare, severe bacterial infection that causes rapidly progressive soft tissue necrosis from the skin to the muscle. Moreover, gas gangrene, Fournier’s gangrene, and necrotizing fasciitis are also included in the NSTI concept [1]. The incidence is reported to be three to four cases per 100,000 persons/year in Europe and the United States [2,3]. The mortality rate in untreated patients is 100% [4]. Even with appropriate treatment, the mortality rate is still high (6.9-29%) [5-8]. NSTI is classified as type 1 (85-90% of NSTI; due to mixed infections with various anaerobic and aerobic organisms) and type 2 (10-15% of NSTI; due to group A beta-hemolytic streptococci [GAS]). In addition, GAS infections of soft tissues are aggressively localized, rapidly progressive, and cause toxic shock syndrome [1,9-11].

NSTI occurs at all ages, with or without comorbidities [7,10,12]. Diabetes mellitus, immunosuppressed status, obesity, alcoholism, peripheral arterial disease, postoperation, and trauma have been reported as risk factors for morbidity. However, no established risk factors have been noted [4,5,7,9,13]. Moreover, no clear diagnostic criteria exist for NSTI. The definitive diagnosis is based on the finding of necrotic changes in soft tissues (color changes, tissue fragility [positive finger test], white cloudy exudate called dishwater, and poor bleeding by skin incision) [7,14]. The gold standard for treating NSTI is a prompt diagnosis, early surgical debridement of necrotic tissue, and antimicrobial therapy [9]. Debridement improves survival [7,15]. Incisions, extensive debridement, and reconstruction are required for treating NSTIs, and plastic surgery is often involved. However, the impact of early surgical intervention of plastic surgeons on the clinical course of NSTIs has not yet been studied. This study investigated the relationship between early plastic surgery intervention and the clinical course of NSTI cases treated at the hospital of this study.

## Materials and methods

Study design

We retrospectively analyzed patients diagnosed with NSTI who were treated at Yokosuka General Hospital Uwamachi between January 2009 and August 2020. The patients were identified based on the data from the medical affairs department. The hospital institutional review board approved the study (approval number: 2018030).

Patients and methods

The NSTI diagnostic criteria were infections with clinically progressive necrosis of the skin and soft tissues. Specifically, the following three criteria were met: (1) gross findings of necrotic changes by skin incision, (2) bacterial presence by wound culture, and (3) performed incision and debridement. Patients who received the best supportive care at the time of the initial treatment and did not receive an active treatment were excluded.

The day of onset was defined as the day of admission or day zero if the onset occurred after admission. Early plastic surgery intervention was defined as “cases in which plastic surgery intervened and performed incision and debridement within 1 day [16].” The rest of the patients were defined as the nonearly intervention group. The end of the treatment period was defined as discharges and the time when inpatient care was no longer necessary as a series of NSTI treatments was completed. The waiting period for rehabilitation and transfer was excluded. Patient background, outcomes (mortality and amputation), and days to the first surgery were compared in the early and nonearly plastic surgery intervention groups. Moreover, the duration of treatment was compared in surviving patients [17].

Statistical analysis

Differences between the two groups were analyzed using Fisher’s direct probability test, Mann-Whitney U test was used for comparison of continuous variables between the two groups, and Spearman’s rank correlation analysis was used for the bivariate correlation coefficient. The significance level was set at <5%. Statistical analysis was performed using STATA software (version 13.1, College Station, TX, USA).

## Results

A total of 30 patients diagnosed with NSTI were treated at the hospital during the study period. Two patients who received the best supportive care at the time of the initial treatment because of their poor systemic conditions due to severe complications were excluded. This study included 28 patients. A total of eight and 20 patients were in the early and nonearly plastic surgery intervention groups (14 in later intervention and six in nonintervention), respectively.

The patients’ characteristics are shown in Table 1. The median age was 64 (0-86) years, with 17 males and 11 females. The clinical outcome was mortality and amputation in seven and six patients (four above-knee and two below-knee amputations), respectively. Consequently, the median treatment duration in the survival group (21 patients) was 58 days (14-251 days). Comorbidities included diabetes (15 patients), vascular disease (12 patients), cardiac disease (11 patients), renal disease (four patients), and liver disease (one patient). All seven mortalities were in the nonearly intervention group. They had pre-existing cardiovascular diseases, untreated diabetes, or malignancy.

**Table 1:** Patient characteristics (n = 28). PRS: plastic and reconstructive surgery

Characteristics	n (%)
Age, years, median (range)	64 (0-86)
Sex	
Male	17 (60.7%)
Female	11 (39.3%)
Comorbidities	
Diabetes	15 (53.6%)
Peripheral vascular disease	12 (42.9%)
Cardiac disease	11 (39.3%)
Kidney disease	4 (14.3%)
Liver disease	1 (3.6%)
Intervention of PRS	22 (78.6%)
Early intervention	8 (28.6%)
Later intervention	14 (50%)
None	6 (21.4%)
Outcomes	
Median length of the treatment period (range)	55 (6–251)
Mortality	7 (25%)
Amputation	6 (21.4%)

The first surgeries were all debridement, except for drainage in three cases and amputation in three cases. The median number of surgeries (incisional drainage and debridement), excluding closed surgery (suturing and skin grafting), was two per patient. Frequent washing and minor debridement were repeated until fine granulation tissues covered the wound surface. Negative pressure wound therapy was applied in 11 cases. Skin grafting was performed in eight cases as a subsequent treatment in extensive raw areas after the infections subsided. All patients received a continuous course of antibiotics in consultation with an infection control team. An appropriate agent was selected with repeated culture tests. The duration of antimicrobial treatment was determined comprehensively based on Labodata and local findings.

The results of the comparison between the early and nonearly intervention groups are shown in Table 2. A difference in the median number of days to the first surgery between the early (zero days) and nonearly (two days) intervention groups was significant (p = 0.002). The median treatment duration in the early (n = 8) and nonearly (n = 20) intervention groups was 44 (14-58 days) and 67 (6-251 days) days, respectively, and it was significantly shorter in the early intervention group (p = 0.027). No significant differences exist in other parameters, including mortality (p = 0.065) and amputation (p = 0.432).

**Table 2:** Comparison between the early and nonearly intervention groups. *: significant p-values.

	Early intervention	Nonearly intervention	P-value
Variable	(n = 8)	(n = 20)	
Sex			
Male	5 (62.5%)	12 (60.0%)	
Female	3 (37.5%)	8 (40.0%)	0.624
Median age (range)	60 (0–82)	65 (28–86)	0.460
Comorbidities			
Diabetes	5 (62.5%)	10 (50.0%)	0.431
Peripheral vascular disease	2 (25.0%)	10 (50.0%)	0.218
Cardiac disease	3 (37.5%)	8 (40.0%)	0.624
Kidney disease	1 (12.5%)	3 (15.0%)	0.682
Liver disease	0	1 (5.0%)	0.714
Days to the first surgery (range)	0	2 (0–16)	0.002*
Median length of the treatment period (range)	44 (14–58)	67 (6–251)	0.027*
Mortality	0	7 (35.0%)	0.065
Amputation	1 (12.5%)	5 (25.0%)	0.432

A comparison of treatment duration in the survival groups is shown in Table 3. The median treatment duration in the early (n = 8) and nonearly (n = 13) intervention groups was 44 (14-58 days) and 82 (35-251 days) days, respectively, which was significantly shorter in the early intervention group (p = 0.003).

**Table 3:** Comparison of treatment duration in the survival groups *: significant p-values.

	Early intervention	Nonearly intervention	P-value
Variable	(n = 8)	(n = 13)	
Sex			
Male	5 (62.5%)	7 (53.8%)	
Female	3 (37.5%)	6 (46.2%)	0.528
Median Age (range)	60 (0–82)	63 (28–86)	0.856
Median length of the treatment period (range)	44 (14–58)	82 (35–251)	0.003*
Amputation	1 (12.5%)	4 (30.8%)	0.344

**Figure 1:**
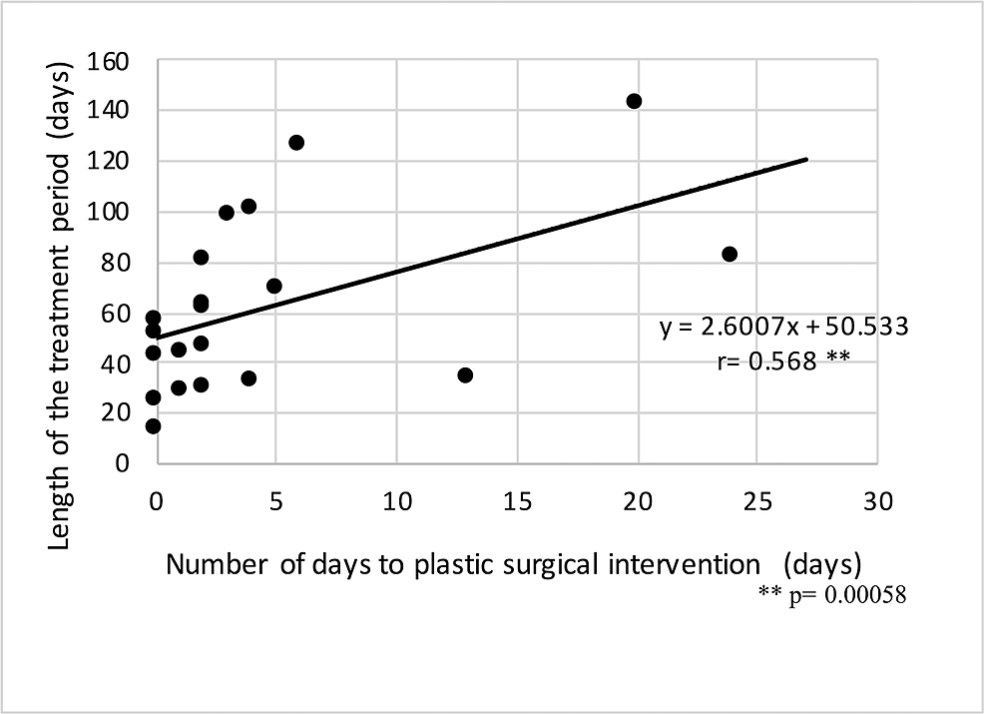
Correlation between the number of days to plastic surgery intervention and the length of the treatment period.

A moderate correlation was noted between the number of days to plastic surgery intervention and the length of the treatment period in the entire plastic surgery intervention group (n = 22; r = 0.568; Figure 1).

## Discussion

This study investigated the relationship between the performance of early plastic surgery intervention and the clinical course of NSTI. The number of days to the first surgery and the length of the treatment period was significantly shorter in the early intervention group compared with the nonearly intervention group. It is believed that this study is the first report to study the effect of the early involvement of plastic surgeons on the clinical course of NSTI.

NSTI is an extremely serious infection with mortality rates of up to 100% when untreated [4]. Even with an appropriate treatment, the mortality rate is high (6.9-29%) [5-8]. The mortality rate in this study was 25%, which is comparable to that reported by various authors. All mortalities in this study were from the nonearly intervention group. An early plastic surgery intervention was not a significant factor in survival (p = 0.065), but it tended to reduce mortality.

The gold standard for treating NSTI is a prompt diagnosis, early and complete surgical debridement of necrotic tissue, and antimicrobial therapy [9]. Early debridement has been reported to improve survival [15]. In a study by McHenry et al., the mean time from admission to surgery was 25 and 90 hours in the survival and mortality groups, respectively. In addition, a study by Sugihara et al. suggested that early surgery within two days of admission in Fournier’s gangrene has a lower mortality rate [18]. Conversely, a report stated that delay in surgery beyond 24 hours of admission is the only factor affecting mortality [7,16], making early surgical intervention important. In this study, no significant difference in mortality existed between patients with and without an early plastic surgery intervention. However, the number of days until the first surgery and the length of the treatment period were significantly shorter in the early intervention group than in the nonearly intervention group. The results suggest that an early plastic surgery intervention can shorten the treatment period.

Furthermore, Anaya and Dellinger along with other researchers mentioned the importance of a “complete” debridement [7,15]. Plastic surgeons are adept at determining the extent of debridement based on intraoperative macroscopic findings as they routinely deal with necrosis from the skin to the muscle. Plastic surgery consistently plays an important role in the initial surgical incision, diagnosis, extensive debridement, and the final reconstruction decision-making for NSTI treatment. Plastic surgeons are accustomed to working with soft tissues of the skin and do not hesitate to make exploring incisions and would be able to distinguish between normal and necrotic tissues at an early stage. The number of days to initial surgery and the length of the treatment period were significantly shorter in the early plastic surgery intervention group in this study. The consistent intervention by plastic surgeons from the beginning of the treatment allows for rapid diagnosis, correct debridement, appropriate decision-making on whether additional surgery is necessary, efficient negative pressure wound therapy, and closed surgery.

Several limitations to this study exist, including the small number of patients. Moreover, it is a single-center, retrospective study. Many patients had underlying diseases that might act as confounding factors when determining the outcomes. Thus, further studies are needed to accumulate more cases.

## Conclusions

This study investigated the effect of early involvement of plastic surgeons on the clinical course of patients with NSTI treated at the hospital. In this study, no statistically significant difference in mortality was found between patients with and without an early plastic surgery intervention. However, the number of days until the first surgery and the length of the treatment period were significantly shorter in the early plastic surgery intervention group than in the nonearly intervention group.
